# Functional and structural biomarkers linked to diabetic retinal neurodegeneration in pre-clinical and early diabetic retinopathy

**DOI:** 10.1186/s12886-025-04564-0

**Published:** 2025-12-10

**Authors:** Jae Yee Ku, Paul C. Knox, Gabriela Czanner, David G. Parry, Simon P. Harding

**Affiliations:** 1 Department of Eye and Vision Science, Institute of Life Course and Medical Sciences, Faculty of Health & Life Sciences, University of Liverpool, William Henry Duncan Building, 6 West Derby Street, Liverpool, L7 8TX UK; 2 St. Paul’s Eye Unit, Liverpool University Hospitals NHS Foundation Trust, Liverpool, L7 8XP UK; 3 School of Electronics and Computer Science, Faculty of Engineering and Physical Science, Faculty of Medicine, University of Southampton, Room 5103, Building 13, Highfield Campus, Southampton, SO17 1BJ UK; 4 Faculty of Informatics and Information Technologies, Slovak University of Technology, Ilkovičova 2, Bratislava, 842 16 Slovakia

**Keywords:** Biomarker, Diabetes, Diabetic retinopathy, Handheld radial shape discrimination, Neurodegeneration, OCT

## Abstract

**Background:**

Diabetic retinal neurodegeneration (DRN) is increasingly recognised as an early and progressive neuronal dysfunction. Despite emerging therapeutic approaches, there are no standardised biomarkers. We aim to investigate functional and structural biomarkers of DRN in people with diabetes (PWD) with no or early diabetic retinopathy (DR).

**Methods:**

Functional measures included handheld radial shape discrimination (hRSD), near and distant visual acuity (VA). Structural changes were evaluated using optical coherence tomography (OCT)-derived retinal thicknesses (Early Treatment Diabetic Retinopathy Study (ETDRS) subfields). Mean thicknesses of the inner and outer ETDRS subfields were averaged to derive inner and outer ring estimates. Pearson’s correlation assessed associations between normally distributed variables. Group differences were analysed using Student’s t-test for continuous data, Chi-squared or Fisher’s exact tests for categorical variables, and one-way ANOVA with Bonferroni-adjusted post hoc tests for multiple groups.

**Results:**

The study included a single eye from 50 healthy participants (HP; Group 1, 55 ± 14y), 26 PWD with no DR (Group 2, 55 ± 14y), and 46 with early DR (Group 3, 57 ± 17y). hRSD and VA were worse in Groups 1 to 3 (all *p* < 0.001). Worse function (measured via hRSD) was associated with inner retinal thinning (*p* < 0.05). Compared to HP, PWD (Groups 2 and 3) showed thinning of the total retina (most subfields), retinal nerve fibre layer (RNFL; outer ETDRS ring), ganglion cell layer (GCL) and inner plexiform layer (IPL) in the inner ring, and outer nuclear layer (ONL) in the central subfield (CSF) (*p* < 0.05). Group 3 also showed GCL and IPL thinning (outer ring) (*p* < 0.05). In 23 PWD followed over 205 ± 109 days, GCL, IPL, and inner nuclear layer (INL) thicknesses decreased (*p* < 0.05).

**Conclusions:**

Functional and structural changes occur early in DR. hRSD and retinal thicknesses are promising biomarkers for DRN. Longitudinal data suggest DRN to be progressive.

**Clinical trial number:**

Not applicable.

## Background

Diabetic retinopathy (DR) is the leading cause of visual loss in working-age adults worldwide and early DR is asymptomatic [1]. Increasing evidence suggests that chronic hyperglycaemia affects the retina before the onset of the microangiopathy, clinically detectable as the classical features of DR. Studies have reported functional deficits in this “pre-clinical” and early stage of the disease compared to healthy participants (HP) [2]. Emerging data support neuroglial dysfunction in early DR, with cross-sectional and longitudinal optical coherence tomography (OCT) findings showing inner retinal thinning [3–7].

Although DR has been traditionally regarded as a microvascular disease, there is growing evidence to support retinal neurodegeneration in the early pathogenesis of DR [3]. This has led to recently coined terms such as diabetic neuroretinopathy or diabetic retinal neurodegeneration (DRN). Significant progress with the detection and treatment of the later stages of DR has been made in recent decades, but there has been less attention to the prevention or treatment of early disease. Neuroprotective strategies have shown promise in early DR and reduced DR progression with fenofibrate [8, 9]. However, there is no general consensus on suitable biomarkers of DRN, nor data on reversibility.

We investigated functional and structural biomarkers of DRN in pre-clinical and early DR. We assessed macular function using the handheld radial shape discrimination (hRSD) test and evaluated retinal structure through OCT-derived thickness measurements. Additionally, we examined the relationship between retinal function and structure by analysing the association between hRSD performance and OCT measurements.

The hRSD test, which evaluates central visual processing and hyperacuity through radial frequency pattern discrimination, is less influenced by media opacity or ageing and is more sensitive to macular pathology than standard acuity tests [10–12]. Hyperacuity enables the detection of spatial misalignments or positional differences with thresholds finer than the diameter of cone photoreceptors, surpassing the eye’s optical resolution by relying on cortical processing and is considered a measure of neural function [12]. Although the hRSD test has been approved by the Food and Drug Administration (FDA) as a means of detecting metamorphopsia in macular disease, its value in DR has yet to be determined, with most hRSD studies on age-related macular degeneration (AMD) [10, 13]. To our knowledge, this is the first study to employ the hRSD test in people with diabetes (PWD) with pre-clinical and early DR. In addition, the few longitudinal studies on DRN have focused mainly on the inner retinal layers [4–7]. This is the first study to longitudinally assess thickness change across all retinal layers in DRN.

## Methods

### Study design

In a prospective observational three-cohort study (HP, PWD with no DR, and PWD with early DR), PWD were assessed at two time points for longitudinal analysis. As this was an observational study, follow-up intervals were determined by the attending ophthalmologist. This study followed the Strengthening the Reporting of Observational Studies in Epidemiology (STROBE) guidelines [14]. One eye per participant was selected for analysis (random selection if both eyes eligible).

### Ethical statement

This study adhered to the Declaration of Helsinki and was approved by the Health Research Authority, Northwest Research Ethics Committee in England (16/NW/0163, V3). All participants received written study information and provided written informed consent.

### Participants

#### Healthy participants

HP (**Group 1**, controls) aged ≥ 18 years were recruited from people accompanying patients and staff at the Royal Liverpool University Hospital (RLUH) to achieve a matched mean age and gender balance between May and July 2017.

#### People with diabetes

PWD, aged ≥ 18 years, newly referred from the Liverpool Diabetic Eye Screening Programme with suspected sight-threatening DR (moderate non-proliferative DR (NPDR), proliferative DR and/or sight-threatening maculopathy were considered for the study [15]. Eligible participants had at least one eye with no maculopathy (clinically and on OCT) and either no DR (**Group 2**, pre-clinical, Early Treatment Diabetic Retinopathy Study (ETDRS) grade 10) or mild NPDR (**Group 3**, early DR, ETDRS grade 20/35). Exclusion criteria included concurrent macular pathology, glaucoma, amblyopia, and cerebral pathologies resulting in visual impairment. No eyes had any macular oedema. PWD were recruited from RLUH between May 2016 and August 2017.

### Variables, data sources and measurement

Participants’ ethnicity, ocular history and general health were recorded. Distance VA was measured using the ETDRS chart (Lighthouse, Precision Vision™) at 4 m with distance correction if worn or pinhole if VA was worse than + 0.10 logMAR, and the best measure was taken. Near VA was tested using the ETDRS 2000 series chart (Lighthouse, Precision Vision™).

hRSD tests were performed using the myVisionTrack (mVT) application (Vital Art & Science LLC, Richardson, Texas) on an Apple iPod Touch, following a previously published protocol and reported as a logMAR score [11]. Near VA and hRSD were tested at 40 cm with the participants’ reading correction, if worn or age-appropriate near correction [16]. All participants underwent slit-lamp biomicroscopy, including fundoscopy examination and macular OCT.

PWD had additional blood pressure (BP) and intraocular pressure (IOP) measurements, and HbA_1c_ results within 3 months of their study visit recorded. PWD attending a second visit repeated all baseline tests for longitudinal analysis.

### OCT methods

**Fig. 1:**
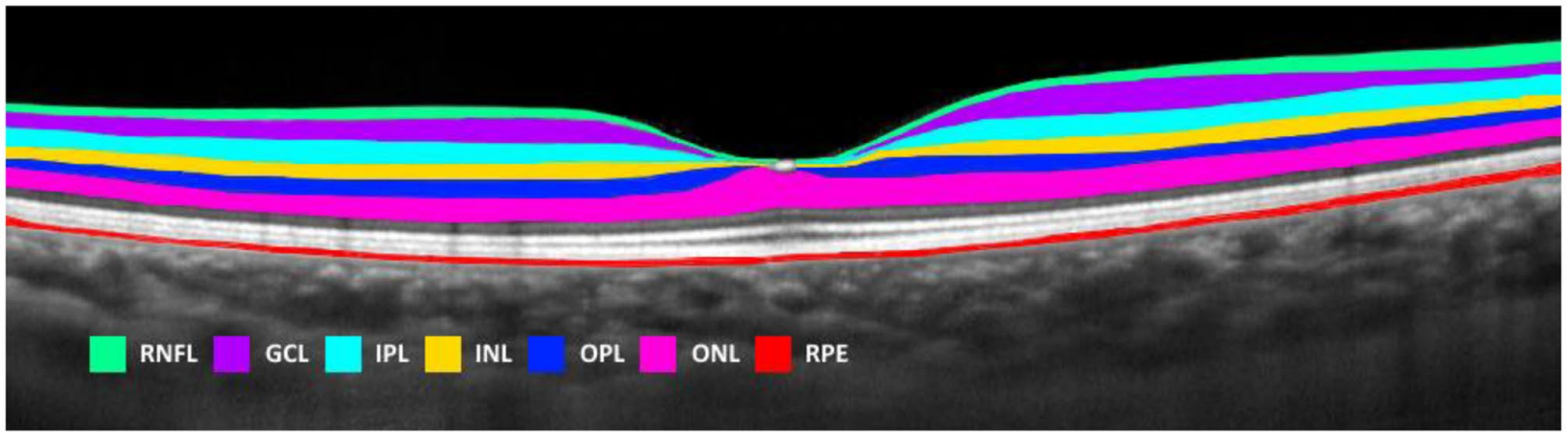
Example Heidelberg Spectralis output of segmented retinal layers from a healthy participant. RNFL = retinal nerve fibre layer, GCL = ganglion cell layer, IPL = inner plexiform layer, INL = inner nuclear layer, OPL = outer plexiform layer, ONL = outer nuclear layer, RPE = retinal pigment epithelium

OCT (Spectralis; Heidelberg Engineering, Heidelberg Eye Explorer version 1.10.0.0, software version 6.8a) was obtained using a 31-line raster scan (30° x 25°) centred on the foveal centre, with eye-tracking and automatic real-time tracking of 16–25 frames. Spectralis auto-segmentation provided initial measurement of total retinal thickness (internal limiting membrane to the retinal pigment epithelium (RPE)/Bruch’s complex), and thicknesses of retinal nerve fibre layer (RNFL), ganglion cell layer (GCL), inner plexiform layer (IPL), inner nuclear layer (INL), outer plexiform layer (OPL), outer nuclear layer (ONL) and RPE across nine ETDRS subfields (Fig. 1). Manual adjustment of segmentation was performed when necessary. OCT evaluation was performed by the first author (JYK) with a subset of central subfield thickness (CST) regraded by another author (DGP) to evaluate inter-grader reliability.

### Study size

The study aimed to compare the groups based on two primary outcomes: mean hRSD and total retinal thickness on OCT. With no prior published work examining both hRSD and OCT data for these groups, there was insufficient information to conduct a formal sample size calculation. Therefore, we used a pragmatic approach, recruiting all eligible participants between May 2016 and August 2017. We estimated that our approach would lead to recruiting approximately 50 HP, 25 PWD with no DR and 50 with early DR.

### Statistical methods

Mean thicknesses of inner and outer ETDRS subfields were averaged to give an inner ring and outer ring estimate. Absolute difference (AD) was the difference between the mean retinal thickness of two groups. Relative difference (RD) was calculated as AD divided by the retinal thickness of the less severe group, expressed as a percentage. Inter-grader reliability was assessed using intra-class correlation (ICC). Pearson’s correlation tested relationships between normally distributed variables. For two-group comparisons, Student’s *t*-test was used for continuous data, Chi-squared for categorical variables, and Fisher’s exact test when expected counts were low. One-way ANOVA with post hoc tests assessed differences across multiple groups, with Bonferroni corrections applied as needed for multiple comparisons. Analyses were performed using Excel (2016), GraphPad Prism (version 8) and SPSS (version 25) with significance set at *p* < 0.05.

## Results

### Description of healthy participants and people with diabetes

50 HP (Group 1), 26 PWD with no DR (Group 2) and 46 PWD with early DR (Group 3) were recruited (Table 1). The groups were matched for age (ANOVA *F* = 0.313, *p* = 0.732) and gender (χ^2^ = 0.103, *p* = 0.950), with most participants identifying as white (Fisher’s exact test, *p* = 0.032; Table 1).

As expected, compared to Group 2, Group 3 were more likely to have type 1 diabetes (*p* = 0.024), longer duration of diabetes (*t* = 4.886, *p* < 0.001), higher HbA_1C_ (*t* = 2.734, *p* = 0.008) and more likely to be on insulin (*p* = 0.001). Both Groups 2 and 3 had moderately elevated BP with no differences in the systolic (*t* = 1.135, *p* = 0.261) and diastolic (*t* = 1.213, *p* = 0.230) BP. All PWD participants had normal IOP.

**Table 1:** Descriptive analyses of eyes from all groups

Group (No. of eyes)	Group 1 (*n* = 50)	Group 2 (*n* = 26)	Group 3 (*n* = 46)	Statistics
Age (years)	55 ± 14	55 ± 14	57 ± 17	*F* = 0.313, *p* = 0.732
Mean ± SD (range)	(22–85)	(23–76)	(20–86)	χ^2^ = 0.103, *p* = 0.950
Gender (No.)				χ^2^ = 0.103, *p* = 0.950
M	26	14	23	
F	24	12	23	
Ethnicityꝉ				Fisher’s exact test, *p* = 0.032
White (%)	48 (96%)	20 (77%)	39 (85%)	
Type of diabetes				Fisher’s exact test, *p* = 0.024
Type 1 (%)	NA*	1 (4%)	12 (26%)	
Type 2 (%)		25 (96%)	34 (73%)	
Duration of known diabetesꝉ (years)	NA*	7.3 ± 4.1	16.6 ± 9.2	*t* = 4.886, *p* < 0.001
HbA_1C_ꝉ (mmol/mol)	NA*	59.2 ± 14.1	72.6 ± 22.5	*t* = 2.734, *p* = 0.008
On insulin (%)	NA*	3 (12%)	23 (50%)	Fisher’s exact test, *p* = 0.001
BP (mmHg)	NA*			
Systolic		144 ± 15	137 ± 24	*t* = 1.135, *p* = 0.261
Diastolic		84 ± 11	79 ± 12	*t* = 1.213, *p* = 0.230

*Not applicable ꝉ Missing data: Ethnicity - Group 3, 1 missing data. Duration of known diabetes - Group 3, 1 missing data. HbA_1c_ - Group 3, 2 missing data

### Inter-grader reliability for macular structure

Given that the positioning of the centre of the ETDRS grid is essential for the accuracy of obtaining subsequent retinal thickness measurements, CST inter-grader reliability was assessed. CST was independently measured in 30 HP and 15 PWD by two graders (JYK and DGP). Agreement was consistently high in HP (ICC 1.000, 95% CI 0.999 to 1.000, *F*_29,29_=3542,432, *p* < 0.001) and PWD (ICC 1.000, 95% CI 1.000 to 1.000, *F*_14,14_=20847,571, *p* < 0.001).

### Macular function via hRSD

**Fig. 2:**
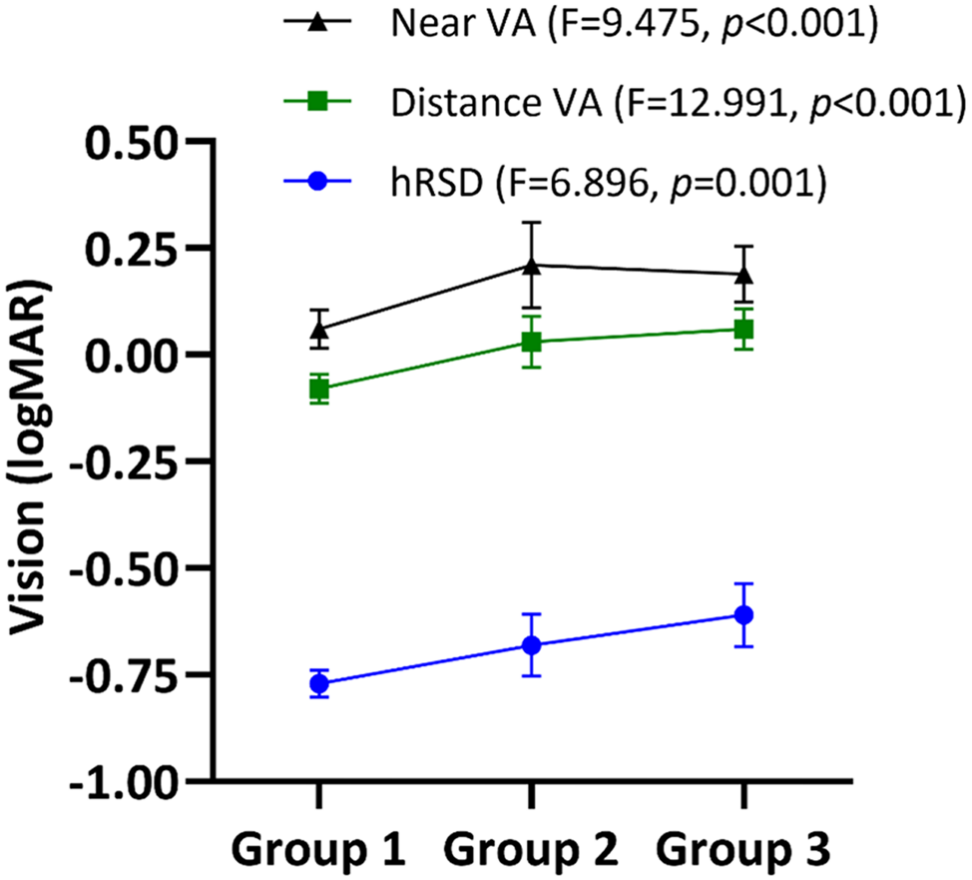
Function of the eyes across three groups in terms of vision and hRSD. Mean (± 95%CI) hRSD threshold, distance and near VA of HP (Group 1), PWD with no DR (Group 2) and PWD with early DR (Group 3) with ANOVA shown. hRSD, distance and near VA data are plotted together for convenience

Eyes in PWD with no or early DR showed reduced visual function compared to HP. hRSD threshold (logMAR mean ± SD Group 1 -0.77 ± 0.11, Group 2 -0.68 ± 0.18, Group 3 -0.61 ± 0.25), distance VA (Group 1 -0.08 ± 0.12, Group 2 + 0.03 ± 0.15, Group 3 + 0.06 ± 0.16) and near VA (Group 1 + 0.06 ± 0.16, Group 2 + 0.21 ± 0.25, Group 3 + 0.19 ± 0.22) was generally worse with increasing disease severity for each of the tests (Fig. 2; see figure for ANOVA results). Inter-group analysis detected significant differences in hRSD threshold between Groups 1 and 3 (*p* < 0.001); for distance VA between Groups 1 and 2 (*p* = 0.006) and Groups 1 and 3 (*p* < 0.001), and near VA between Groups 1 and 2 (*p* = 0.008) and 1 and 3 (*p* = 0.006).

When all the groups were combined, there was a significant reduction in hRSD threshold associated with thinning of the total retina in the inner (*r* = 0.21, *p* = 0.022) and outer rings (*r* = 0.21, *p* = 0.023). In addition, there was also a significant reduction in hRSD threshold associated with thinning of the RNFL in the outer ring (*r* = 0.18, *p* = 0.049), and GCL and IPL, both the inner (GCL *r* = 0.23, *p* = 0.013; IPL *r* = 0.24, *p* = 0.009) and outer rings (GCL *r* = 0.19, *p* = 0.040; IPL *r* = 0.19, *p* = 0.038).

### Macular structure via OCT

#### Total retinal thickness by ETDRS subfields

**Fig. 3:**
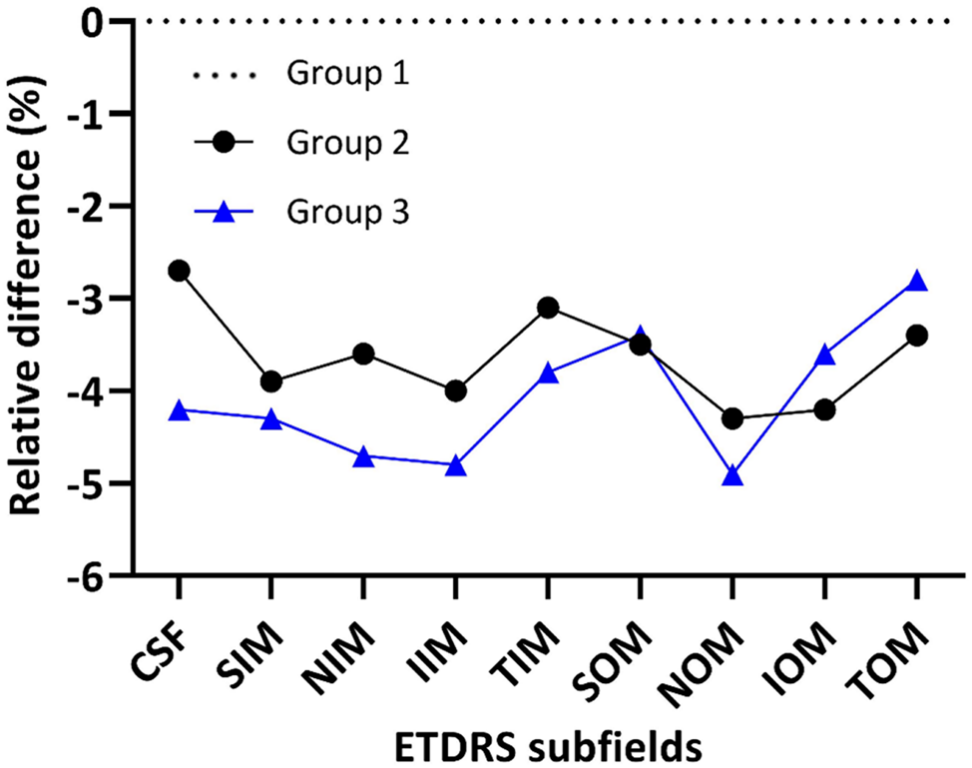
Relative difference (RD; %) of total retinal thickness between Group 1 (dotted black line), Group 2 (black line) and Group 3 (grey line). RD was calculated as the difference between the mean retinal thickness of PWD and Group 1, divided by the retinal thickness of Group 1, expressed as a percentage. CSF = central subfield, SIM = superior inner macula, NIM = nasal inner macula, IIM = inferior inner macula, TIM = temporal inner macula, SOM = superior outer macula, NOM = nasal outer macula, IOM = inferior outer macula, TOM = temporal outer macula

Compared to Group 1, mean total retinal thicknesses were significantly reduced in both Groups 2 and 3 across all subfields (range − 2.7 to -4.9%; all *p* < 0.05) (Fig. 3; Table 2) except for Group 2 in the central subfield (CSF). Thinning was generally more apparent in Group 3, particularly in the central and inner subfields, suggesting progression with DR (Fig. 3).

**Table 2:** Total retinal thicknesses (mean ± SD, µm) by ETDRS subfields in all groups

ETDRS subfields*	Group 1 (mean ± SD, µm)	Group 2 (mean ± SD, µm)	Group 3 (mean ± SD, µm)	Differences			
				Group 2 vs. 1		Group 3 vs. 1	
				AD (µm)	RD (%)	AD (µm)	RD (%)
CSF	282.7 ± 23.9	275.2 ± 18.6	270.8 ± 25.7	-7.5	-2.7	-11.9	-4.2
SIM	345.6 ± 14.6	332.0 ± 12.7	330.6 ± 18.2	-13.6	-3.9	-15.0	-4.3
NIM	347.9 ± 15.8	335.4 ± 15.9	331.7 ± 18.6	-12.5	-3.6	-16.2	-4.7
IIM	342.4 ± 13.3	328.7 ± 14.9	325.8 ± 16.4	-13.7	-4.0	-16.6	-4.8
TIM	331.9 ± 12.3	321.7 ± 14.3	319.4 ± 16.1	-10.2	-3.1	-12.5	-3.8
SOM	300.3 ± 13.7	289.7 ± 10.6	290.2 ± 16.0	-10.6	-3.5	-10.1	-3.4
NOM	317.6 ± 13.2	304.0 ± 14.9	301.9 ± 18.4	-13.6	-4.3	-15.7	-4.9
IOM	288.6 ± 11.8	276.6 ± 12.1	278.2 ± 15.5	-12.0	-4.2	-10.4	-3.6
TOM	282.1 ± 12.1	272.5 ± 9.6	274.2 ± 11.8	-9.6	-3.4	-7.9	-2.8

Differences in mean retinal thickness compared to HP expressed in absolute difference (AD; µm) and relative difference (RD; %). *ETDRS subfields: CSF = central subfield, SIM = superior inner macula, NIM = nasal inner macula, IIM = inferior inner macula, TIM = temporal inner macula, SOM = superior outer macula, NOM = nasal outer macula, IOM = inferior outer macula, TOM = temporal outer macula

#### Differential effect on retinal layers

**Fig. 4:**
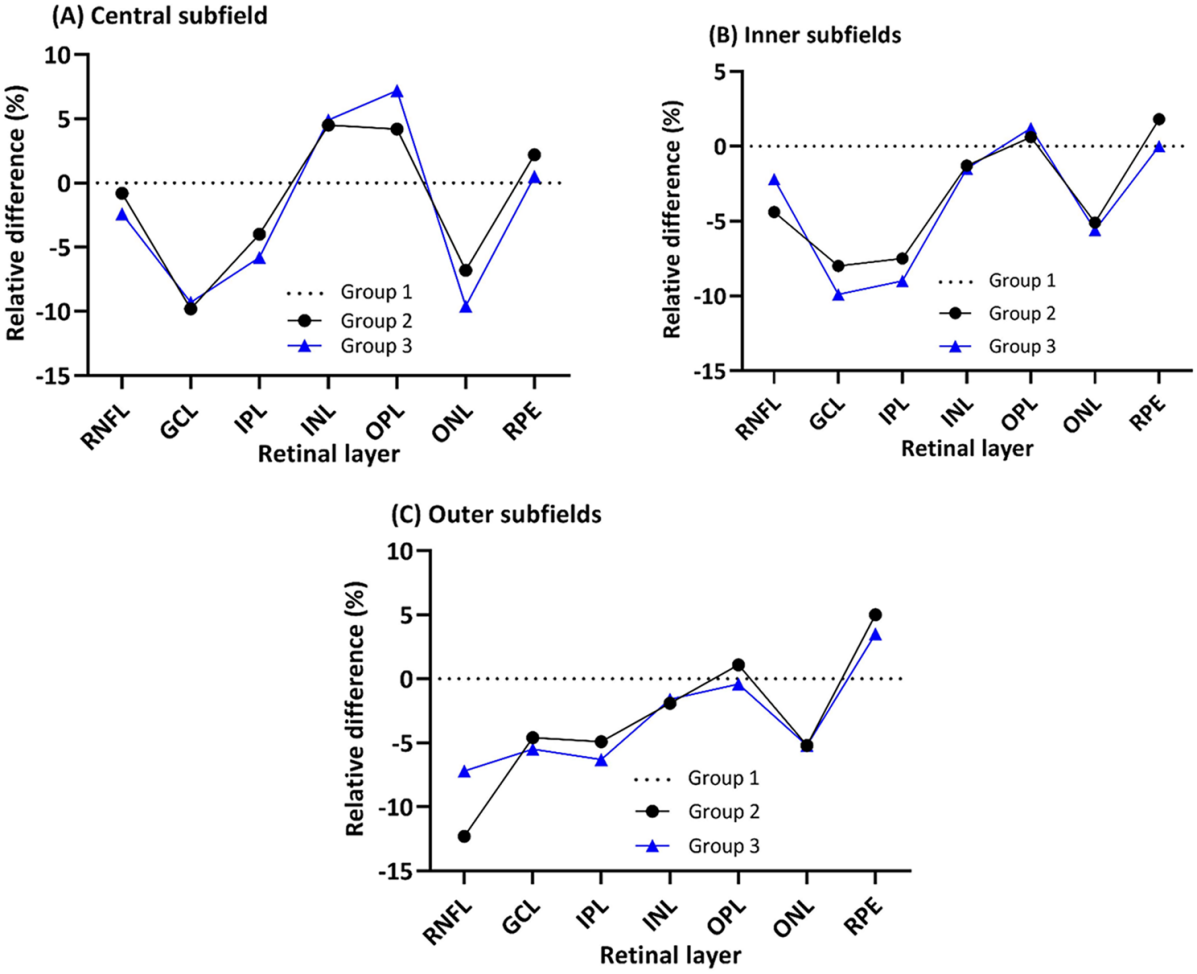
Relative difference (RD; %) of different retinal layers between Group 1 (dotted black lines), Group 2 (black lines) and Group 3 (blue lines) in the central subfield (**A**), inner ring (**B**) and outer ring (**C**). RNFL = retinal nerve fibre layer, GCL = ganglion cell layer, IPL = inner plexiform layer, INL = inner nuclear layer, OPL = outer plexiform layer, ONL = outer nuclear layer, RPE = retinal pigment epithelium

Compared to Group 1, Groups 2 and 3 showed inner retinal thinning across all subfields (RNFL, -0.8 to -12.3%; GCL, -4.6 to -9.9%; IPL, -4.0 to -9.0%) except for slight INL thickening in the CSF (+ 4.5 to + 5.0%) (Fig. 4; Table 3). These findings were statistically significant for RNFL in the outer ring (*p* < 0.05) and GCL and IPL in the inner ring (all *p* < 0.01). For Group 3, this was also significant for GCL (*p* = 0.015) and IPL (*p* = 0.001) in the outer ring (Table 4).

In the outer retina, compared to Group 1, there was ONL thinning in Groups 2 and 3 in all subfields (-5.1 to -9.7%), and this was significant in the CSF (all *p* < 0.05). Interestingly, compared to Group 1, Groups 2 and 3 had thicker RPE in most subfields (+ 0.6% to + 5.0%), and this was significant in the outer ring (*p* < 0.05) (Table 4).

**Table 3:** Retinal thicknesses of retinal layers in the central subfield, inner and outer ETDRS rings

Subfield	Group 1 (mean ± SD, µm)	Group 2 (mean ± SD, µm)	Group 3 (mean ± SD, µm)	Differences			
				Group 2 vs. 1		Group 3 vs. 1	
				AD (µm)	RD (%)	AD (µm)	RD (%)
**Central Subfield**							
Total	282.7 ± 23.9	275.2 ± 18.6	270.8 ± 25.7	-7.5	-2.7	-11.9	-4.2
RNFL	13.3 ± 2.0	13.2 ± 2.3	13.0 ± 2.6	-0.1	-0.8	-0.3	-2.3
GCL	16.3 ± 4.6	14.7 ± 4.0	14.8 ± 3.9	-1.6	-9.8	-1.5	-9.2
IPL	22.4 ± 4.1	21.5 ± 3.9	21.1 ± 4.0	-0.9	-4.0	-1.3	-5.8
INL	19.8 ± 6.3	20.7 ± 5.9	20.8 ± 6.1	0.9	4.5	1.0	5.0
OPL	26.5 ± 5.6	27.6 ± 6.6	28.4 ± 5.6	1.1	4.2	1.9	7.2
ONL	94.1 ± 9.7	87.7 ± 11.5	85.0 ± 10.8	-6.4	-6.8	-9.1	-9.7
RPE	18.0 ± 2.1	18.4 ± 2.2	18.1 ± 2.0	0.4	2.2	0.1	0.6
Inner Ring*							
Total	342.0 ± 13.5	329.4 ± 13.9	326.9 ± 16.5	-12.6	-3.7	-15.1	-4.4
RNFL	22.9 ± 1.9	21.9 ± 2.0	22.4 ± 1.8	-1.0	-4.4	-0.5	-2.2
GCL	51.3 ± 4.4	47.2 ± 4.8	46.2 ± 5.6	-4.1	-8.0	-5.1	-9.9
IPL	42.4 ± 3.0	39.2 ± 3.2	38.6 ± 3.6	-3.2	-7.5	-3.8	-9.0
INL	39.1 ± 3.1	38.7 ± 2.8	38.6 ± 3.0	-0.4	-1.0	-0.5	-1.3
OPL	33.4 ± 3.9	33.6 ± 4.5	33.8 ± 3.3	0.2	0.6	0.4	1.2
ONL	70.9 ± 8.4	67.3 ± 8.6	66.9 ± 7.7	-3.6	-5.1	-4.0	-5.6
RPE	16.3 ± 1.4	16.6 ± 1.3	16.3 ± 1.4	0.3	1.8	0	0
Outer Ring*							
Total	297.2 ± 11.9	285.4 ± 10.5	286.3 ± 14.5	-11.8	-4.0	-10.9	-3.7
RNFL	37.3 ± 4.6	32.7 ± 4.3	34.6 ± 4.7	-4.6	-12.3	-2.7	-7.2
GCL	34.7 ± 3.2	33.1 ± 2.8	32.8 ± 3.7	-1.6	-4.6	-1.9	-5.5
IPL	28.8 ± 2.3	27.4 ± 1.9	26.9 ± 2.6	-1.4	-4.9	-1.9	-6.6
INL	32.0 ± 1.9	31.3 ± 2.0	31.5 ± 2.0	-0.7	-2.2	-0.5	-1.6
OPL	27.7 ± 1.8	28.0 ± 2.4	27.6 ± 1.7	0.3	1.1	-0.1	-0.4
ONL	57.8 ± 6.1	54.8 ± 6.5	54.8 ± 6.2	-3.0	-5.2	-3.0	-5.2
RPE	14.1 ± 1.1	14.8 ± 1.0	14.6 ± 1.3	0.7	5.0	0.5	3.5

Retinal thickness expressed in absolute difference (AD; µm) and relative difference (RD; %) compared to Group 1. * Thicknesses for inner and outer subfields are presented as means of the 4 segments in each ring

**Table 4:** Statistically significant differences in retinal layer thickness in the central, inner and outer ETDRS rings

Retinal layer	Group/mean retinal thickness (µm)		Absolute difference between groups (µm)	Relative difference between groups (%)	*P*
**Central Subfield**					
Full	Group 1/282.7	Group 3/270.8	-11.9	-4.2	0.047
ONL	Group 1/94.1	Group 2/87.7	-6.4	-6.8	0.041
	Group 1/94.1	Group 3/85.0	-9.1	-9.7	< 0.001
**Inner Ring**					
Full	Group 1/342.0	Group 2/329.4	-12.6	-3.7	0.002
	Group 1/342.0	Group 3/326.9	-15.1	-4.4	< 0.001
GCL	Group 1/51.3	Group 2/47.2	-4.1	-8.0	0.003
	Group 1/51.3	Group 3/46.2	-5.1	-9.9	< 0.001
IPL	Group 1/42.4	Group 2/39.2	-3.2	-7.5	< 0.001
	Group 1/42.4	Group 3/38.6	-3.8	-9.0	< 0.001
**Outer Ring**					
Full	Group 1/297.2	Group 2/285.4	-11.8	-4.0	0.001
	Group 1/297.2	Group 3/286.3	-10.9	-3.7	< 0.001
RNFL	Group 1/37.3	Group 2/32.7	-4.6	-12.3	< 0.001
	Group 1/37.3	Group 3/34.6	-2.7	-7.2	0.015
GCL	Group 1/34.7	Group 3/32.8	-1.9	-5.5	0.015
IPL	Group 1/28.8	Group 3/26.9	-1.9	-6.6	0.001
RPE	Group 1/14.1	Group 2/14.8	0.7	5.0	0.044

Absolute differences (AD), relative differences (RD) and *p*-values from post-hoc comparisons are shown, following the significant ANOVA comparison at 5% level of significance

### Longitudinal results

Of the 72 PWD, 23 remained as either having no DR (*n* = 8) or early DR (*n* = 15) during their second visit, and were available for longitudinal analysis. Retinal thickness from all subfields in Groups 2 and 3 was combined for analysis using paired Student’s *t*-tests.

The mean ± SD interval between visits was 205 ± 109 days. Their mean ± SD distance VA, near VA and hRSD threshold remained stable between visits (distance VA, visit 1 0.07 ± 0.15, visit 2 0.05 ± 0.15, paired *t*-test 1.352, *p* = 0.191; near VA, visit 1 0.17 ± 0.21, visit 2 0.11 ± 0.16, *t* = 1.725, *p* = 0.100; hRSD visit 1 -0.67 ± 0.14, visit 2 -0.68 ± 0.13, *t* = 0.151, *p* = 0.881). HbA_1c_ decreased significantly from 70.2 ± 26.1 to 63.7 ± 16.1 mmol/mol between visits (*t* = 2.321, *p* = 0.037). Significant GCL, IPL and INL thinning was observed between visits (Table 5).

**Table 5:** Retinal thickness in PWD with no or early DR in visits 1 and 2

Thicknessꝉ	Visit 1 (µm) Mean ± SD	Visit 2 (µm) Mean ± SD	Paired t-test	p*
RNFL	26.83 ± 2.64	26.89 ± 2.80	0.150	0.882
GCL	37.73 ± 3.56	37.27 ± 3.84	2.523	**0.020**
IPL	31.98 ± 2.48	31.61 ± 2.69	2.517	**0.020**
INL	33.89 ± 1.92	32.96 ± 1.11	3.129	**0.005**
OPL	29.90 ± 2.38	29.31 ± 1.95	1.923	0.068
ONL	63.96 ± 6.80	64.37 ± 6.69	0.780	0.444
RPE	15.98 ± 1.23	16.29 ± 1.26	1.776	0.090

Retinal thickness in different layers in all ETDRS subfields * Statistically significant results are shown in bold. ꝉThere is no missing data

## Discussion

This study revealed that hyperacuity reduction and retinal thinning occur early in DRN, before clinically detectable microvasculopathy. Compared to HP, PWD without DR showed clinically meaningful declines in hRSD and distance VA (+ 0.09, + 0.11, respectively), equivalent to 1 ETDRS line. PWD with early DR showed further reductions of approximately half a line (+ 0.16, + 0.14, respectively) compared to HP. Near VA also declined by approximately 1.5 lines in both groups (no DR + 0.15, early DR + 0.13). While two small studies have examined hRSD changes in more severe DR, our study is the first to report hRSD deficits in pre-clinical and early DR [10, 17]. Our findings align with those of Ahmed et al., who found a 2-line reduction in distance VA in 44 PWD with mild NPDR compared to 30 controls [18].

We observed reduced hRSD in PWD compared to HP, with performance progressively worsening across disease stages. Similarly, Wang et al. reported declining hRSD with increasing DR severity in a smaller cohort of PWD with more advanced DR (*n* = 36) [10]. Unlike Wang et al., who focused on eyes with macular thickening (CST ≥ 240 μm), we found a significant correlation between hRSD decline and thinning of total and inner retinal layers. Collectively, these findings suggest that hRSD may correlate with retinal thickness in DR.

Glassman et al. recently identified low luminance VA, contrast sensitivity, flicker electroretinogram and objective field analyser as promising tools for DR screening and research [2]. We propose adding hRSD to this list, given its affordability, ease of use, and potential for remote deployment. Notably, hRSD is impaired in early DR and opens up the potential to study pre-clinical DR as a distinct stage in disease progression.

We observed significant total retina thinning in PWD with no or early DR compared to HP across most subfields. In the CSF, inner and outer rings (approximately equivalent to the fovea, parafovea and perifovea areas, respectively), there was evidence of thinning of the GCL and IPL. These differences were statistically significant for the inner (Group 1 vs. both Groups 2 and 3) and the outer rings (Group 1 vs. Group 3), but not the CSF. This is most likely due to the GCL and IPL being thinner in the fovea compared to the parafoveal and perifoveal areas (Fig. 1). Montesano et al. also reported total retina thinning in PWD with no DR (total retina − 3.47 μm, GCL − 1.04 μm, IPL − 1.89 μm) compared to HP, but there was no RNFL difference [19]. The more pronounced thinning in our study may be due to the longer duration of diabetes in our participants (7.3 ± 4.1 years vs. 2 months).

Our segmentation analysis identified novel findings in the outer retina, while most DRN studies have focused on inner retina layers [3–7]. We observed thinning of the ONL in PWD with no and early DR compared to HP, which was significant in the CSF. ONL has a large component of photoreceptor nuclei. Photoreceptors are metabolically active cells and susceptible to oxidative stress and metabolic dysregulation in diabetes [20]. Diabetic animal models have generally confirmed ONL thinning, but results in human OCT studies have been variable [20–22]. Another notable outer retinal finding was increased RPE thickness in PWD, most prominently in the outer ring. Similar results were reported by Ferreira et al. [23]. However, the significance of this observation remains unclear and merits further investigation.

A key strength of our study is the inclusion of PWD with pre-clinical and early DR, who are typically not referred to hospital eye services. This group may represent a critical phase in the disease process before visible microvascular changes appear. Our study also benefits from a well-aged and gender-matched control group, and a PWD sample (*n* = 72), comparable to previous DRN studies [4–6]. However, our longitudinal group was relatively small, and the follow-up period was limited to approximately six months.

We found inner retina and ONL thinning, adding to emerging evidence of progressive thinning [4]. However, due to the complexities in accurately measuring retinal layers in more severe DR, it is unknown if retinal thinning is linear, accelerates with more severe disease, or stabilises with improved glycaemic control. The 5–10% inner retinal and ONL thinning correlated to a visual decline of 1-1.5 ETDRS lines. While this effect size is modest, it is biologically meaningful. Our PWD had reasonably good distance vision (no DR + 0.03 ± 0.15, early DR + 0.06 ± 0.16) and were probably visually asymptomatic, but were significantly worse than our HP group (-0.08 ± 0.12).

Among the few longitudinal DRN studies, only Sohn et al.’s is directly comparable with ours, due to similar OCT protocols [4–7]. They followed 45 PWD with no or mild DR over 73 months and reported significant RNFL loss of 0.25 μm/year and combined GCL and IPL loss of 0.29 μm/year [4]. Our study found significant GCL (0.46 μm) and IPL (0.37 μm) loss over approximately 6 months. Our findings suggest a greater annual loss of GCL and IPL compared to their study. Several factors may explain this discrepancy. Firstly, PWD in our study (55–57 years) were older than in Sohn et al. (31 years). Secondly, we had a smaller number of PWD (*n* = 23) with a shorter duration of follow-up (205 ± 109 days), which may limit the precision of the estimates as reflected by the relatively wide SD.

**Fig. 5:**
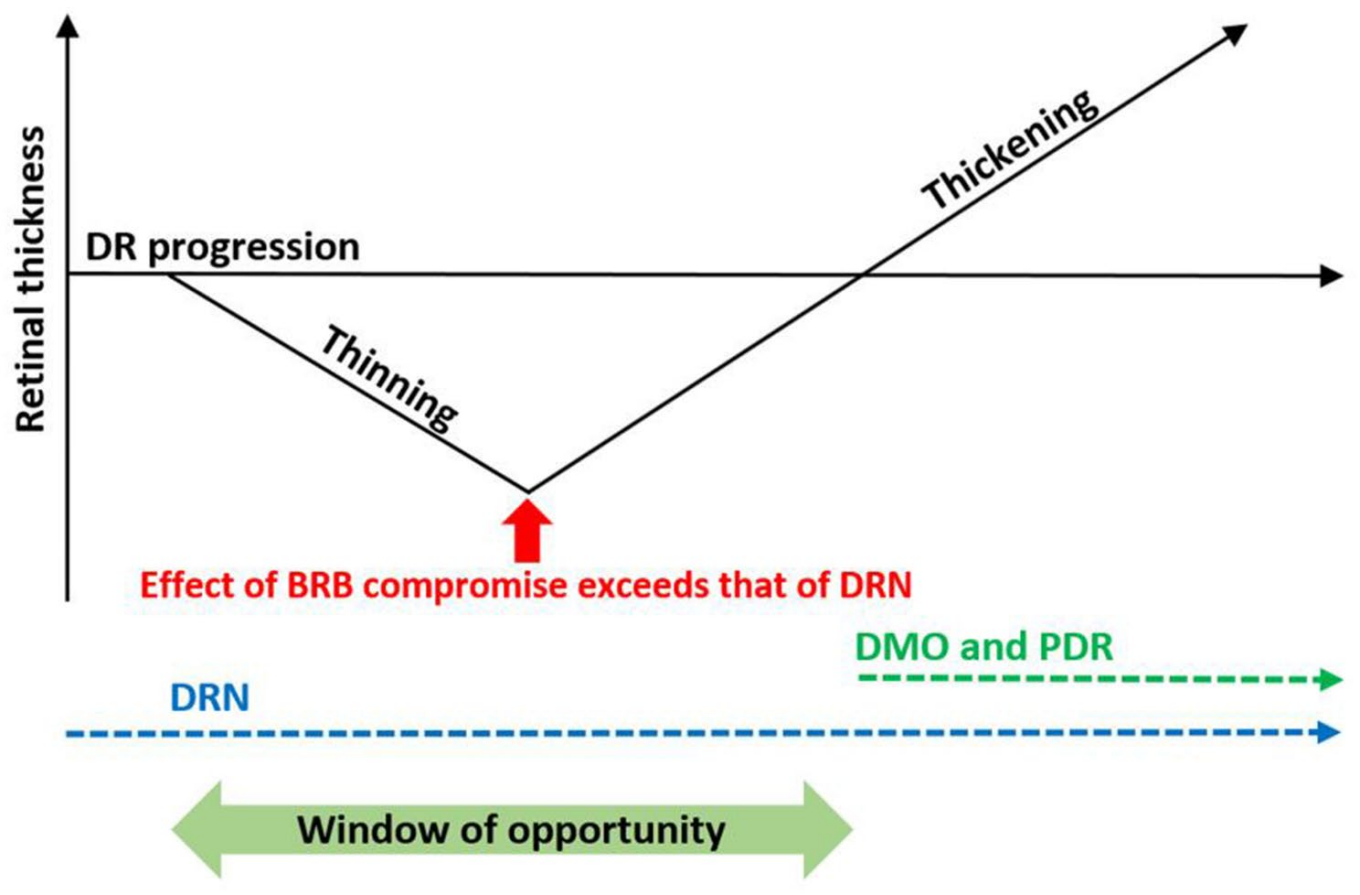
As DR progresses (horizontal axis), retinal thinning (vertical axis) can occur in DRN. When the blood retinal barrier (BRB) compromise on the retina exceeds that of DRN (red arrow), retinal thickness increases. With increasing BRB compromise, diabetic macular oedema (DMO) and proliferative diabetic retinopathy (PDR) can become apparent, leading to retinal thickening. The window of opportunity for neuroprotective agents to work in DRN (dotted blue line) should be before the onset of DMO and PDR (dotted green line)

One complexity in using retinal thickness as a biomarker for DRN is that vascular leakage can increase retinal thickness, potentially masking retinal thinning in more severe DR. Bandello et al. studied 194 eyes with mild NPDR and found retinal thinning only when eyes with subclinical and clinical macular oedema were analysed separately [24]. They noted thicker INL and OPL, possibly due to extracellular fluid accumulation caused by alterations in the inner blood retinal barrier (BRB). This suggests that total retinal thickening occurs when the fluid-induced thickening of the INL and OPL surpasses the retinal thinning caused by DRN in the inner retinal layers and the ONL. We present in Fig. 5a timeline of retinal thickness in DR, introducing the preclinical and early phase of retinal thinning before the well-recognised thickening develops at the time of BRB breakdown and subsequent progression to DMO. DRN is an underlying pathogenic factor throughout. We believe that there is a “window of opportunity” for neuroprotection. The EUROCONDOR [8, 25] and LENS [9] studies suggested that neuroprotective agents such as brimonidine, somatostatin and fenofibrate may slow DRN progression, but further clinical translation is needed [26].

## Conclusions

In conclusion, we identified both functional and structural changes consistent with DRN in pre-clinical and early DR. We observed hRSD and VA deficits with inner retinal and ONL thinning. Our evidence supports hRSD and retinal thickness as biomarkers for DRN. We have added to the notion of pre-clinical DR as a specific phase in the natural history of diabetes whereby DRN occurs before any clinically visible microvasculopathy. Lastly, we developed the concept of DRN as an underlying pathogenic factor in DR progression.

## Data Availability

Available from the corresponding author upon reasonable request.

## Abbreviations

AD: Absolute difference
AMD: Age-related macular degeneration
BP: Blood pressure
BRB: Blood retinal barrier
CSF: Central subfield
CST: Central subfield thickness
DRN: Diabetic retinal neurodegeneration
DR: Diabetic retinopathy
ETDRS: Early Treatment Diabetic Retinopathy Study
FDA: Food and Drug Administration
GCL: Ganglion cell layer
hRSD: handheld radial shape discrimination
HP: Healthy participants
ICC: Intra-class correlation
IOP: Intraocular pressure
INL: Inner nuclear layer
IPL: Inner plexiform layer
IIM: Inferior inner macula
IOM: Inferior outer macula
mVT: myVisionTrack
NIM: Nasal inner macula
NOM: Nasal outer macula
NPDR: Non-proliferative DR
OCT: Optical coherence tomography
ONL: Outer nuclear layer
OPL: Outer plexiform layer
PWD: People with diabetes
RD: Relative difference
RNFL: Retinal nerve fibre layer
RPE: Retinal pigment epithelium
RLUH: Royal Liverpool University Hospital
STROBE: Strengthening the Reporting of Observational Studies in Epidemiology
SIM: Superior inner macula
SOM: Superior outer macula
TIM: Temporal inner macula
TOM: Temporal outer macula
VA: Visual acuity
